# Fibrinogenase and Direct Thrombin Inhibitor for Injection in the Treatment of Acute Ischemic Stroke

**DOI:** 10.3390/jcm15083112

**Published:** 2026-04-19

**Authors:** Chao Zhang, Haibing Liao, Zhiying Wang, Fanshu Zhao, Wei Yue

**Affiliations:** 1 Tianjin Key Laboratory of Cerebral Vascular and Neurodegenerative Diseases, Department of Neurology, Clinical College of Neurology, Neurosurgery, and Neurorehabilitation, Huanhu Hospital Affiliated to Tianjin Medical University, Tianjin 300350, China; czhanghh@163.com (C.Z.); liaohaibing1991@126.com (H.L.); wangzhiying202302@163.com (Z.W.); zhaofs1101@163.com (F.Z.); 2 Department of Biomedical Engineering, Tianjin University, Tianjin 300072, China; 3 Department of Neurology, Tianjin Huanhu Hospital, 6 Jizhao Rd., Tianjin 300350, China

**Keywords:** fibrinogenase, direct thrombin inhibitors, stroke, treatment, thromboproprotein

## Abstract

**Introduction**: Fibrinogenase and direct thrombin inhibitors (DTIs) have emerged as potential therapeutic agents for stroke, leveraging distinct mechanisms of action. However, different treatment strategies impact on the risk of stroke recurrence and in-hospital neurological progression remains inadequately characterized. **Methods**: We conducted a study enrolling patients with acute ischemic stroke (AIS) between 1 April 2022, and 1 July 2023. Data on demographics, comorbidities, clinical characteristics, and laboratory parameters were collected. Prognostic assessment was based on the incidence of recurrent stroke and in-hospital neurological deterioration, functional outcome (measured by the modified Rankin Scale, mRS), and neurological status (assessed by the National Institutes of Health Stroke Scale, NIHSS). **Results**: Among the 618 patients included in the final analysis, 187 received fibrinogenase, 127 received a DTI, and 304 served as controls. The overall stroke recurrence rate was 16.3%, with incidences of 23.0% (43/187) in the fibrinogenase group, 23.6% (30/127) in the DTI group, and 9.2% (28/304) in the control group. Neurological deterioration during admission occurred in 40 patients (6.5%), with rates of 4.3%, 12.6%, and 5.3% in the fibrinogenase, DTI, and control groups, respectively. Subgroup analysis revealed that fibrinogenase administration in patients with elevated plasma thromboproprotein (TpP) levels was associated with reduced recurrence rates and improved clinical outcomes. **Discussion**: Our findings preliminarily indicate that fibrinogenase and DTI therapies are more frequently employed in patients at higher risk of recurrence or progression. Specifically, fibrinogenase injection appears to suggest potential as an efficacious and well-tolerated treatment option for improving prognosis in AIS patients with elevated TpP levels.

## 1. Introduction

Stroke, characterized by sudden neurological deficits, represents a major global health burden. In high-income countries, up to one in five individuals may be affected during their lifetime, while in low-income countries, this figure approaches one in two, making stroke the second leading cause of death worldwide [1]. Ischemic stroke (IS) constitutes approximately 70% to 80% of all stroke cases and is a leading cause of mortality and long-term disability globally [2,3]. The current standard of care for acute ischemic stroke relies on intravenous thrombolysis and endovascular thrombectomy [4]. While these interventions have revolutionized stroke care, their clinical utility is substantially hindered by stringent eligibility criteria. The narrow therapeutic time window excludes a majority of patients who present late or with unclear symptom onset [5]. Furthermore, the evidence supporting these aggressive interventions in patients presenting with mild but disabling neurological deficits remains limited and controversial, often leading to therapeutic conservatism in a significant patient subgroup; even when applied, these treatments are not always successful; incomplete recanalization or early re-occlusion can occur, leaving residual thrombus burden that perpetuates ischemic risk [6].

In response to these limitations, the therapeutic landscape for AIS has broadened to include a range of adjunctive pharmacological agents. Among these, injectable pharmacological agents such as fibrinogenase (a defibrinogenating enzyme derived from snake venom that directly degrades fibrinogen) and direct thrombin inhibitor (DTI) have gained attention as novel adjuvant therapies [7]. The mechanistic rationale for their use is compelling: by promoting endogenous fibrinolysis and simultaneously inhibiting the coagulation cascade, these drugs may enhance the clearance of microvascular thrombi and prevent thrombus propagation [8,9]. This dual action offers the theoretical advantage of improving microcirculatory perfusion and reducing the risk of early recurrent ischemic events without a commensurate, prohibitive increase in hemorrhagic transformation [5,9]. Despite their growing use, the comparative effectiveness and real-world utilization patterns of agents with distinct mechanisms of action remain poorly characterized. In addition, the underlying cause of stroke may also play a role in determining patient prognosis. And there are special pathophysiology, prognosis, and clinical features of lacunar strokes [10]. In lacunar ischemic strokes, recurrent lacunes are the most frequent cause of subcortical dementia and, in cardioembolic stroke, is the most important predictor of mortality; stroke recurrence is different in ischemic stroke subtypes [11]. Further evidence is needed to evaluate how different treatment strategies influence stroke prognosis, thereby enabling more tailored prevention and monitoring approaches. To address this gap, our study analyzed data from a large prospective clinical cohort to describe contemporary treatment patterns in acute ischemic stroke (AIS) and to assess the impact of different therapeutic approaches on the risk of stroke recurrence and in-hospital neurological progression.

## 2. Method

### 2.1. Study Design and Population

A total of 630 patients diagnosed with acute ischemic stroke (AIS) who were admitted to Tianjin Huanhu Hospital between 1 April 2022, and 1 July 2023, were included in this study. The study was approved by the hospital’s Institutional Review Board. The inclusion criteria were as follows: (1) age 18–80 years; (2) meeting the diagnostic criteria for AIS (all patients underwent magnetic resonance imaging (MRI) with diffusion-weighted imaging (DWI) to confirm the diagnosis); (3) providing informed consent to participate in this study and being willing and able to complete the 12-month follow-up assessment; and (4) having complete data for relevant variables. Patients were excluded if they met any of the following criteria: (1) diagnosis of hemorrhagic stroke, brain trauma, hemangioma, or tumor; (2) undergoing surgery (e.g., decompressive craniotomy), or being pregnant or lactating; (3) having severe symptoms, such as a Glasgow Coma Scale score ≤ 12, severe organ dysfunction (e.g., heart, liver, or kidney), or a life expectancy of less than one year; (4) requiring oral anticoagulants or having an acute hemorrhagic tendency or coagulation disorders; or (5) having taken other fibrinolytic drugs or medications affecting fibrinogen, thrombus fibrin, or thrombin formation within the preceding week.

### 2.2. Definition of Confounders and Intermediary Variables

We collected data on health conditions, comorbidities, clinical characteristics, and blood indicators. NIHSS scores were categorized as mild (0–1), moderate (2–4), moderate-to-severe (5–15), and severe (≥16). Blood samples were drawn following hospital admission and prior to the administration of any fibrinogenase or direct thrombin inhibitor therapy for hematological analysis. Plasma thromboprotein (TpP), analyzed as a continuous variable, was also examined after categorization into two groups (by median split) and four groups (by quartile split). Patients were followed up one year after discharge to assess their clinical outcomes and quality of life. The follow-up assessments were conducted via telephone interviews and/or face-to-face visits, depending on the patient’s availability and preference. The primary endpoint was defined as stroke recurrence within the 1-year follow-up period. Secondary endpoints included: (1) the modified Rankin Scale (mRS) score at 1 year after randomization; (2) the National Institutes of Health Stroke Scale (NIHSS) score at discharge; and (3) neurological deterioration, defined as an increase in the NIHSS score of ≥2 points during hospitalization. The safety outcome was the occurrence of hemorrhagic events during treatment and throughout the 1-year follow-up.

### 2.3. Statistical Analysis

Statistical analysis was performed using SPSS version 26, with statistical significance set at *p* < 0.05. Continuous variables are described as median and interquartile range. Differences in categorical variables between groups were compared using the chi-square test, while the Kruskal–Wallis H test was used for continuous variables (the Shapiro–Wilk test was applied to assess data normality). To further explore the impact of the TpP levels and different treatment strategies on the prognosis of AIS patients, a logistic regression model with an interaction term was constructed. For subgroup analysis comparing the two medication groups, the chi-square test and the Mann–Whitney U test were used.

## 3. Results

### 3.1. Baseline Characteristics of the Study Population

A total of 630 patients were initially screened for this study. Among them, 11 patients were excluded due to loss to follow-up (missing primary outcome data), and 1 patient was excluded due to incomplete baseline characteristics (missing all demographic and laboratory data). Loss to follow-up was attributed to two main reasons: 6 patients could no longer be contacted, while others declined further participation. The demographic and clinical characteristics of the patients receiving different therapeutic regimens are presented in Table 1. The handling of missing data and the results of the Shapiro–Wilk test are provided in Supplementary Tables S1 and S2. Data on all medications administered during hospitalization were extracted from electronic medical records, with analysis focusing on the use of injectable fibrinogenase and DTI. Of the 618 included patients, 187 were treated with fibrinogenase, 127 with DTI, and 304 were in the control group. Drinking habits differed significantly among the treatment groups (fibrinogenase group, 143 (76.5%); DTI group, 77 (60.6%); and control group, 217 (71.4%); *p* = 0.010). No significant differences were observed among the three groups in terms of the remaining demographics, comorbidities, clinical characteristics. The baseline median NIHSS score was 3 (IQR 1–6) in the fibrinogenase group, 2 (IQR 1–6) in the DTI group, and 2 (IQR 1–5.75) in the control group. The baseline median TpP level was 3.70 (IQR 2.37–6.12) in the fibrinogenase group, 3.87 (IQR 2.54–4.88) in the DTI group, and 4.68 (IQR 2.52–7.70) in the control group. Apart from the differences noted above, no significant differences were observed among the groups for the other blood indicators.

### 3.2. Comparison of Clinical Outcomes

**Figure 1:**
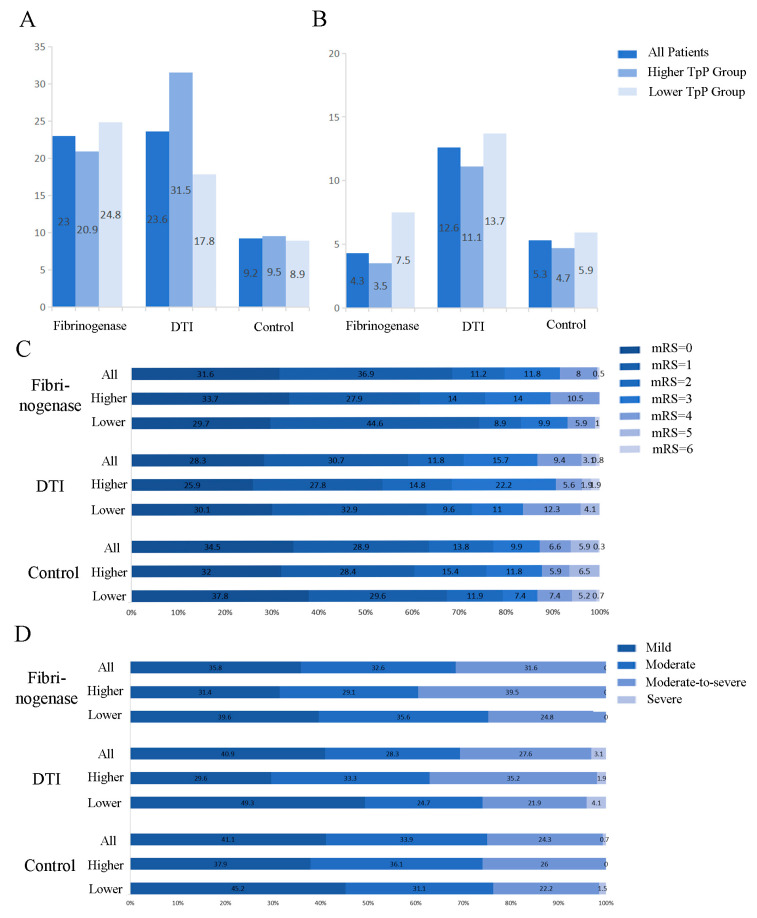
Efficacy outcomes. (**A**) The incidence of stroke recurrence within the 1-year follow-up period. (**B**) The incidence of neurological deterioration during hospitalization. (**C**) Distribution of modified Rankin Scale (mRS) scores within the 1-year follow-up period. (**D**) Distribution of the National Institutes of Health Stroke Scale (NIHSS) score at discharge. DTI: Direct Thrombin Inhibitor. Plasma thromboprotein (TpP) was categorized into two groups by median split. A score of 0 on the mRS indicates no symptoms, a score of 1 indicates no clinically significant disability, a score of 2 indicates slight disability, a score of 3 indicates moderate disability, a score of 4 indicates moderately severe disability, a score of 5 indicates severe disability and a score of 6 indicates death. NIHSS scores were categorized as mild (0–1), moderate (2–4), moderate-to-severe (5–15), and severe (≥16).

Among the 618 acute stroke patients, stroke recurrence occurred in 101 patients (16.3%): 43 (23.0%) in the fibrinogenase group, 30 (23.6%) in the DTI group, and 28 (9.2%) in the control group (*p* < 0.001, Figure 1A). Neurological deterioration during admission occurred in 40 patients (6.5%), with rates of 4.3%, 12.6%, and 5.3% in the fibrinogenase, DTI, and control groups, respectively (*p* = 0.006, Figure 1B). Three deaths were recorded during follow-up (two due to pulmonary infection and one due to large hemispheric infarction). The distribution of the mRS scores at 1 year is shown in Figure 1C. The median discharge NIHSS score was 2 in all groups (fibrinogenase: IQR 1–6; DTI: IQR 1–5; control: IQR 1–4.75). Outcomes stratified by discharge NIHSS score are presented in Figure 1D. No hemorrhagic events were recorded in any patient.

### 3.3. Subgroup Analyses

TpP levels differed significantly among the three groups (*p* = 0.002). To evaluate whether there was an association between the TpP levels and treatment modality, an interaction term between TpP concentration (as a continuous variable) and treatment group (categorical: fibrinogenase, DTI, control) was included in the logistic regression model. A significant interaction was observed between TpP concentration and treatment modality with respect to the risk of stroke recurrence (*p* = 0.014). The interaction coefficients indicated that the effect of TpP concentration differed significantly between the fibrinogenase group and the control group (*p* = 0.027), but not between the DTI group and the control group (*p* = 0.322). The interaction between TpP concentration and treatment modality did not reach statistical significance for the risk of progressive stroke (*p* = 0.069). Although a trend toward effect modification was observed, the absence of a significant interaction suggests that the association between TpP concentration and the risk of progressive stroke was largely consistent across treatment groups (the fibrinogenase group, *p* = 0.067; the DTI group, *p* = 0.058).

In the subgroup with higher TpP levels (defined by median split), the stroke recurrence rate was 20.9% in the fibrinogenase group, compared to 31.5% in the DTI group and 9.5% in the control group (higher-TpP-level group, *p* < 0.001; lower-TpP-level group, *p* = 0.004). Conversely, the rate of neurological deterioration was substantially lower in the fibrinogenase group than in the other two groups (higher-TpP-level group, *p* = 0.127; lower-TpP-level group, *p* = 0.064; Figure 1A,B). A similar trend was observed in the subgroup analysis based on TpP quartiles (Stroke recurrence: Group 1, *p* = 0.011; Group 2, *p* = 0.046; Group 3, *p* = 0.012; Group 4, *p* < 0.001. Neurological deterioration: Group 1, *p* = 0.010; Group 2, *p* = 0.460; Group 3, *p* = 0.500; Group 4, *p* = 0.215). No significant differences were found in mRS or NIHSS scores across the subgroups (Figure 1C,D). We grouped patients into quartiles based on their TpP levels and evaluated the differences in mRS scores and NIHSS scores among the groups. The final results showed no significant inter-group differences (Supplementary Tables S3 and S4).

## 4. Discussion

AIS remains a major global threat to human health and survival. We all got 618 patients included in the analysis, 187 were treated with fibrinogenase, 127 with DTI, and 304 were in the control group. The rate of stroke recurrence was 16.3% (101/618), and 40 patients (6.5%) had neurological deterioration during admission. Previous studies examining trends in stroke recurrence and mortality over the past two decades reported a decline until around 2005, after which rates stabilized at approximately 12% (range 10–15%) [7]. Consistent with these earlier observations, this was significantly higher in both active treatment groups compared with controls. The incidence of in-hospital progressive stroke was 6.5%. No statistically significant differences were observed across treatment groups in stroke recurrence rates, mRS scores, or discharge NIHSS scores, but a higher proportion of patients treated with fibrinogenase achieved favorable functional outcomes. And patients with high baseline TpP levels that received fibrinogenase exhibited a sustained reduction in the risk of both recurrent ischemic events and in-hospital neurological deterioration, in the subgroup analyses.

As an observational study, our investigation focused on fibrinogenase and DTI as key therapeutic variables to assess their real-world impact on short-term neurological progression and long-term stroke recurrence, by leveraging data from a large prospective cohort. Fibrinogenase, a zinc metalloproteinase, is proposed to improve neurological deficits and functional outcomes in AIS patients through three principal mechanisms [12,13,14,15]. First, fibrinogenase selectively degrades soluble fibrin monomers and polymers, the key structural components of nascent and mature thrombi, thereby directly reducing thrombus burden. Second, it potentiates the activity of endogenous tissue plasminogen activator (tPA), amplifying the body’s native fibrinolytic capacity without the systemic lytic state associated with pharmacological doses of tPA. Third, it cleaves circulating plasminogen into soluble fragments, generating active plasmin derivatives that further contribute to fibrin degradation. Argatroban, a representative DTI and an arginine derivative, competitively inhibits thrombin at its active site, thereby reducing fibrin deposition and potentially limiting thrombus propagation in patients with early neurological deterioration [16,17,18]. In our cohort, it is noteworthy that the DTI-treated group exhibited a significantly higher baseline incidence of progressive stroke compared to patients receiving other therapeutic modalities. This finding likely reflects a real-world clinical preference for selecting argatroban in precisely those patients presenting with or at high risk for symptom progression, rather than indicating any causative role of the drug itself. At one-year follow-up, no statistically significant differences were observed across treatment groups in stroke recurrence rates, mRS scores, or discharge NIHSS scores. This aligns with prior reports indicating that intravenous DTIs are not associated with superior outcomes in acute stroke [16,19,20]. In contrast, a higher proportion of patients treated with fibrinogenase achieved favorable functional outcomes (mRS score ≤2). This observed efficacy may be attributed to the potent fibrinolytic activity of fibrinogenase against pre-formed thrombi, which could translate into more complete recanalization and salvage of penumbral tissue. Furthermore, this benefit must be contextualized within the baseline characteristics of our study population. The majority of enrolled patients presented with mild neurological deficits, as evidenced by the fact that 66% of the cohort had an admission NIHSS score of 4 or less. In such a population, the safety profile of fibrinogenase—which carries a lower intrinsic risk of hemorrhagic transformation compared to more aggressive reperfusion strategies—may allow for a more favorable risk–benefit balance. These findings underscore the complexity of therapeutic decision making in AIS. While fibrinogenase may offer advantages in promoting favorable functional recovery, particularly in patients with mild to moderate stroke severity, the role of DTI therapy may be more appropriately reserved for specific clinical scenarios, such as acute neurological deterioration or suspected ongoing thrombus propagation.

Further stratification of patients with elevated TpP levels revealed that the benefits of fibrinogenase therapy were not only maintained but were notably amplified in this subgroup. Specifically, patients presenting with high baseline TpP levels who received fibrinogenase exhibited a sustained reduction in the risk of both recurrent ischemic events and in-hospital neurological deterioration. Moreover, this therapeutic advantage was also observed in relation to long-term functional outcomes, with a significantly higher proportion of these patients achieving complete neurological independence—defined as an mRS score of 0 at the one-year follow-up—compared to those who did not receive the therapy. TpP, formally known as total fibrinogen and fibrin degradation products, represents a heterogeneous mixture of cross-linked fibrin derivatives and serves as a primary substrate for plasmin-mediated fibrinolysis, and has been linked to increased mortality and adverse outcomes in acute coronary syndrome [12,15]. The pathophysiological link is thought to involve persistent thrombotic activity and impaired endogenous fibrinolysis, leading to microvascular obstruction and exacerbated ischemic injury [14]. Unlike more transient markers of coagulation activation, TpP exhibits a prolonged half-life in the circulation, providing a more stable and integrated measure of ongoing thrombus formation and degradation [21]. Consequently, elevated TpP levels may serve as a sensitive indicator of a hypercoagulable state and ongoing fibrin turnover, capturing a snapshot of active prothrombotic processes that other markers might miss. Previous prospective work has established that fibrinogenase injection significantly reduces TpP levels [14]. Thus, for AIS patients with elevated TpP, early fibrinogenase administration by directly addressing the underlying procoagulant imbalance holds the potential to mitigate the elevated risks associated with hypercoagulability, ultimately translating into improved survival and superior functional recovery.

Several limitations of this study should be acknowledged. First, due to its observational design, treatment allocation was subject to clinical bias, with fibrinogenase and/or DTI more frequently administered in severe cases, and there is no randomization. Second, although numerical differences were observed between groups, some did not reach statistical significance, and stroke subtypes were not analyzed separately in our study. Therefore, future larger prospective studies are warranted to assess the efficacy of different treatment approaches according to stroke subtype, which may help guide targeted therapeutic strategies and ultimately improve patient prognosis. Finally, as our cohort predominantly consisted of patients with mild neurological deficits (low NIHSS scores), the generalizability of conclusions to severe stroke populations remains to be validated.

Furthermore, the comparative effectiveness of various treatment approaches for acute stroke patients, especially those with severe stroke, remains to be established. Further large-scale studies are warranted to better evaluate treatment outcomes and inform clinical decision making in this population. The therapeutic advantages of fibrinogenase therapy in patients with high TpP levels remain to be established, and large-scale, multicenter, long-term prospective randomized controlled trials are warranted to address this evidence gap.

In summary, our analysis preliminarily indicates that fibrinogenase and DTI therapies are more commonly utilized in patients at high risk of recurrence or progression. Specifically, fibrinogenase administration in individuals with elevated TpP levels was associated with reduced recurrence rates and improved clinical outcomes. These results suggest that fibrinogenase injection may offer an efficacious and well-tolerated treatment option for improving prognosis in selected AIS patients, and will probably maximize benefits for the greatest number of patients with stroke.

## Figures and Tables

**Table 1:** Baseline characteristics of patients.

Variables	Fibrinogenase		DTI		Control Group		*p* Value
	N	%	N	%	N	%	
All	187	30.3	127	20.6	304	49.2	
Demographics							
Age	65 (57, 71)		63 (55, 71)		65 (57, 73)		0.227
Male	136	72.7	91	71,7	213	70.1	0.812
Smoking	107	57.2	57	67.6	165	54,3	0.087
Drinking	143	76.5	77	60.6	217	71.4	0.010
Co-morbidities							
Hypertension	160	85.9	109	85.8	239	78.6	0.072
Diabetes	123	65.8	75	59.1	203	66.8	0.296
CAD	52	27.8	47	37.0	99	32.6	0.221
Clinical characteristics							
Baseline NIHSS score	3 (1, 6)		2 (1, 6)		2 (1, 5.75)		0.659
Mild	59	31.6	38	29.9	109	35.9	0.669
Moderate	64	34.2	44	34.6	102	33.6	
Moderate-to-severe	63	33.7	42	33.1	89	29.3	
Severe	1	0.5	3	2.4	4	1.3	
Blood indicators							
TpP	3.70 (2.37, 6.12)		3.87 (2.54, 4.88)		4.68 (2.52, 7.70)		0.002
Platelet	224 (189, 262)		215 (192, 253)		224 (188.50, 260.25)		0.756
PCT	0.23 (0.20, 0.26)		0.23 (0.20, 0.26)		0.23 (0.19, 0.27)		0.552
PT	11.40 (10.90, 11.80)		11.40 (10.90, 11.80)		11.40 (10.90, 11.80)		0.740
APTT	24.85 (22.42, 27.50)		25.60 (22.20, 29.20)		24.40 (21.48, 27.50)		0.177
TT	17.40 (16.90, 18.00)		17.60 (17.00, 18.40)		17.30 (16.90, 18.00)		0.069
FIB	2.76 (2.35, 3.39)		2.82 (2.43, 3.40)		2.82 (2.41, 3.39)		0.842
INR	0.96 (0.92, 1.00)		0.97 (0.91, 1.02)		0.97 (0.92, 1.00)		0.781

DTI: direct thrombin inhibitor; CAD: Coronary Artery Disease; TpP: plasma thromboproprotein; PCT: Platelet Crit; PT: Prothrombin Time; APTT: Activated Partial Thromboplastin Time; TT: Thrombin Time; FIB: fibrinogen; INR: International Normalized Ratio. Note: Percentages may not sum to 100% due to rounding.

## Data Availability

The original data and materials will be freely available to any scientist wishing to use them for non-commercial purposes. They can be obtained by contacting the corresponding author via email.

## Supplementary Materials

The following supporting information can be downloaded at https://www.mdpi.com/article/10.3390/jcm15083112/s1, Table S1: The missing data of characteristics; Table S2: The Shapiro–Wilk test results; Table S3: Distribution of modified Rankin Scale (mRS) scores within the 1-year follow-up period; Table S4: Distribution of the National Institutes of Health Stroke Scale (NIHSS) score at discharge.

## Abbreviation

IS	ischemic stroke
MRI	magnetic resonance imaging
DWI	diffusion-weighted imaging
TpP	thromboprotein
the mRS score	the modified Rankin scale score
the NIHSS score	the National Institutes of Health stroke scale score
tPA	tissue plasminogen activator
