# Supplementary material for: Fibrinogenase and Direct Thrombin Inhibitor for Injection in the Treatment of Acute Ischemic Stroke
Source: J Clin Med. 2026 Apr 19;15(8):3112. doi: 10.3390/jcm15083112 (PMC13117420; doi:10.3390/jcm15083112)
Supplement: Supplementary file 1 [file jcm-15-03112-s001.zip › Support Table S1.pdf]

Support Table S1: The missing data of characters.

Variables	All	Fibrinogenase	DTI	Control Group
**Blood indicators**				
TpP	0			
Platelet	42	8	8	26
PCT	45	9	8	28
PT	106	27	28	51
APTT	106	27	28	51
TT	106	27	28	51
FIB	106	27	28	51
INR	106	27	28	51

DTI: Direct Thrombin Inhibitor; TpP: plasma thromboproprotein; PCT: Platelet Crit; PT: Prothrombin Time; APTT: Activated Partial Thromboplastin Time; TT: Thrombin Time; FIB: Fibrinogen; INR: International Normalized Ratio;
