# Supplementary material for: Fibrinogenase and Direct Thrombin Inhibitor for Injection in the Treatment of Acute Ischemic Stroke
Source: J Clin Med. 2026 Apr 19;15(8):3112. doi: 10.3390/jcm15083112 (PMC13117420; doi:10.3390/jcm15083112)
Supplement: Supplementary file 1 [file jcm-15-03112-s001.zip › Support Table S2.pdf]

Support Table S2: The result of Shapiro-Wilk test.

Variables	**Fibrinogenase**		**DTI**		**Control Group**	
	**w**	**p**	**w**	**p**	**w**	**p**
Age	0.985	0.090	0.969	P=0.019	0.992	P=0.228
Baseline NIHSS score	0.869	<0.001	0.839	<0.001	0.828	<0.001
**Blood indicators**						
TpP	0.904	<0.001	0.813	<0.001	0.953	<0.001
Platelet	0.965	0.001	0.955	0.002	0.959	<0.001
PCT	0.967	0.001	0.972	0.037	0.964	<0.001
PT	0.854	<0.001	0.682	<0.001	0.958	<0.001
APTT	0.985	0.085	0.851	<0.001	0.879	<0.001
TT	0.990	<0.001	0.369	<0.001	0.256	<0.001
FIB	0.901	<0.001	0.909	<0.001	0.903	<0.001
INR	0.842	<0.001	0.664	<0.001	0.954	<0.001

DTI: Direct Thrombin Inhibitor; TpP: plasma thromboproprotein; PCT: Platelet Crit; PT: Prothrombin Time; APTT: Activated Partial Thromboplastin Time; TT: Thrombin Time; FIB: Fibrinogen; INR: International Normalized Ratio;
