# Supplementary material for: Fibrinogenase and Direct Thrombin Inhibitor for Injection in the Treatment of Acute Ischemic Stroke
Source: J Clin Med. 2026 Apr 19;15(8):3112. doi: 10.3390/jcm15083112 (PMC13117420; doi:10.3390/jcm15083112)
Supplement: Supplementary file 1 [file jcm-15-03112-s001.zip › Support Table S3.pdf]

Support Table S3: Distribution of modified Rankin Scale (mRS) scores at 1-year follow-up period.

	N	mRS=0	mRS=1	mRS=2	mRS=3	mRS=4	mRS=5	mRS=6
**Fibrinogenase**	**All**	59	69	21	22	15	0	1
	**Higher TpP group**	29	24	12	12	9	0	0
	**Lower TpP group**	30	45	9	10	6	0	1
	**Group 1**	17	20	5	6	2	0	0
	**Group 2**	13	25	4	4	4	0	1
	**Group 3**	14	15	7	6	6	0	0
	**Group 4**	15	9	5	6	3	0	0
**DTI**	**All**	36	39	15	20	12	4	1
	**Higher TpP group**	14	15	8	12	3	1	1
	**Lower TpP group**	22	24	7	8	9	3	0
	**Group 1**	9	11	3	2	5	1	0
	**Group 2**	13	13	4	6	4	2	0
	**Group 3**	10	13	5	7	3	0	1
	**Group 4**	4	2	3	5	0	1	0
**Control**	**All**	105	88	42	30	20	18	1
	**Higher TpP group**	54	48	26	20	10	11	0
	**Lower TpP group**	51	40	16	10	10	7	1
	**Group 1**	30	16	11	6	3	6	1
	**Group 2**	21	24	5	4	7	1	0
	**Group 3**	21	24	4	9	3	7	0
	**Group 4**	33	24	22	11	7	4	0

DTI: Direct Thrombin Inhibitor. Plasma thromboprotein (TpP) was categorized into two groups by median split and four groups by quartiles. Group 1 was defined as the 0-25th percentile, Group 2 as the 25th-50th percentile, Group 3 as the 50th-75th percentile, Group 2 as the 75th-100th percentile. A score of 0 on the mRS

indicates no symptoms, a score of 1 indicates no clinically significant disability, a score of 2 indicates slight disability, a score of 3 indicates moderate disability, a score of 4 indicates moderately severe disability, a score of 5 indicates severe disability and a score of 6 indicates death. NIHSS scores were categorized as mild (0 – 1), moderate (2 – 4), moderate-to-severe (5 – 15), and severe (⩾16).
