# Supplementary material for: Fibrinogenase and Direct Thrombin Inhibitor for Injection in the Treatment of Acute Ischemic Stroke
Source: J Clin Med. 2026 Apr 19;15(8):3112. doi: 10.3390/jcm15083112 (PMC13117420; doi:10.3390/jcm15083112)
Supplement: Supplementary file 1 [file jcm-15-03112-s001.zip › Support Table S4.pdf]

Support Table S4: Distribution of the National Institutes of Health Stroke Scale (NIHSS) score at discharge.

	N	Mild	Moderate	Moderate-to-severe	Severe
Fibrinogenase	All	67	61	59	0
	Higher TpP group	27	25	34	0
	Lower TpP group	40	36	25	0
	Group 1	22	15	13	0
	Group 2	18	21	12	0
	Group 3	16	14	18	0
	Group 4	11	11	16	0
DTI	All	52	36	35	4
	Higher TpP group	16	18	19	1
	Lower TpP group	36	18	16	3
	Group 1	15	8	6	2
	Group 2	21	10	10	1
	Group 3	15	15	8	1
	Group 4	1	3	11	0
Control	All	125	103	74	2
	Higher TpP group	64	61	44	0
	Lower TpP group	61	42	30	2
	Group 1	29	27	16	1
	Group 2	32	15	14	1
	Group 3	32	22	14	0
	Group 4	32	39	30	0

DTI: Direct Thrombin Inhibitor. Plasma thromboprotein (TpP) was categorized into two groups by median split and four groups by quartiles. Group 1 was defined as the 0-25th percentile, Group 2 as the 25th-50th percentile, Group 3 as the 50th-75th percentile, Group 2 as the 75th-100th percentile. A score of 0 on the mRS indicates no symptoms, a score of 1 indicates no clinically significant disability, a score of 2 indicates slight disability, a score of 3 indicates moderate disability, a score of 4 indicates moderately severe disability, a score of 5 indicates severe disability and a score of 6 indicates death. NIHSS scores were categorized as mild (0-1), moderate (2-4), moderate-to-severe (5-15), and severe (≥16).
